# Analysis of the association of EPHB6, EFNB1 and EFNB3 variants with hypertension risks in males with hypogonadism

**DOI:** 10.1038/s41598-018-32836-x

**Published:** 2018-09-27

**Authors:** Tao Wu, Bi-Qi Zhang, John Raelson, Yu-Mei Yao, Huan-Dong Wu, Zao-Xian Xu, Francois-christophe Marois-blanchet, Muhammad Ramzan Tahir, Yujia Wang, W. Edward Bradley, Hongyu Luo, Jiangping Wu, Jian-Zhong Sheng, Shen-Jiang Hu

**Affiliations:** 1 Institute of Cardiology, First Affiliated Hospital, College of Medicine, Zhejiang University, Hangzhou, 310003 China; 2 Research Centre, Centre hospitalier de l’Université de Montréal (CHUM), Montreal, Quebec, H2X 0A9 Canada; 3 Department of Cardiology, Third Affiliated Hospital of Zhejiang Chinese Medical University, Hangzhou, 310005 China; 4 Children’s Hospital, College of Medicine, Zhejiang University, Hangzhou, Zhejiang, 310003 China; 5 Nephrology Service, Centre hospitalier de l’Université de Montréal (CHUM), Montreal, Quebec, H2X 0A9 Canada; 6 Department of Pathology and Physiopathology, College of Medicine, Zhejiang University, Hangzhou, 310005 China

## Abstract

Several members of the EPH kinase family and their ligands are involved in blood pressure regulation, and such regulation is often sex- or sex hormone-dependent, based on animal and human genetic studies. *EPHB6* gene knockout (KO) in mice leads to hypertension in castrated males but not in un-manipulated KO males or females. To assess whether this finding in mice is relevant to human hypertension, we conducted a human genetic study for the association of *EPHB6* and its two ligands, *EFNB1* and *EFNB3*, with hypertension in hypogonadic patients. Seven hundred and fifty hypertensive and 750 normotensive Han Chinese patients, all of whom were hypogonadic, were genotyped for single nucleotide polymorphisms (SNPs) within the regions of the genes, plus an additional 50 kb 5′ of the genes for *EPHB6*, *EFNB1* and *EFNB3*. An imputed insertion/deletion polymorphism, *rs35530071*, was found to be associated with hypertension at *p*-values below the *Bonferroni*-corrected significance level of 0.0024. This marker is located 5′ upstream of the *EFNB3* gene start site. Previous animal studies showed that while male *EFNB3* gene knockout mice were normotensive, castration of these mice resulted in hypertension, corroborating the results of the human genetic study. Considering the significant associations of *EFNB3* SNPs with hypertension in hypogonadic males and supporting evidence from castrated *EFNB3* KO mice, we conclude that loss-of-function variants of molecules in the *EPHB6* signaling pathway in the presence of testosterone are protective against hypertension in humans.

## Introduction

EPH kinases are receptor tyrosine kinases. They are divided into A and B subfamilies according to sequence homology^1^. EPH ligands are cell surface molecules ephrins (EFN), which are also divided into A and B subfamilies, according to how they anchor on the cell surface (glycosylphosphatidylinositol anchoring for EFNAs and transmembrane anchoring for EFNBs)^1,2^. The interactions between EPHs and EFNs are promiscuous, although in general EPHA subfamily members preferably interact with EFNAs, and EPHBs interact with EFNBs^1,2^.

EPHs and EFNs are critical in the development and function of the central nervous system^3^, immune system^4–16^, and digestive system^17^, and in different tissues and processes, such as bone homeostasis^18,19^, angiogenesis^20^, etc.^20–23^.

We have recently reported that EPHB6, EPHB4, EFNB1, EFNB2 and EFNB3 are novel regulators in blood pressure (BP)^24–28^. While deletion of EPHB6, EFNB1 and EFNB3 results in increased BP^24–26^, EPHB4 and EFNB2 deletion leads to lower BP^27,28^ in mice. Such BP regulation is often sex- and/or sex hormone-specific^24–28^. Thus, these molecules likely function as yin and yang in maintaining BP homeostasis, with sex hormones as modifiers. Our human genetic studies corroborate the findings in mice. We have found that five single nucleotide polymorphisms (SNPs) in the 3′ region of *EFNB*2 gene are significantly associated with hypertension for male but not female patients with type 2 diabetes, and the coding (minor) allele of these SNPs are protective against hypertension in male Caucasians^28^. In the same cohort, two SNPs in the 3′ region of *EFNB3* gene are associated with hypertension risks in male but not female Caucasians^29^. In a separate cohort of International Blood Pressure Consortium, we have revealed that a SNP in *GRIP1* gene, whose protein product is in the *EFNB3* signaling pathway, is significantly associated with diastolic BP of 69,395 individuals^30^. Taken together, these mouse and human studies confirm that EPHs and EFNs are previously unknown BP regulators, and they deserve our attention in our effort to control the epidemic health issue of hypertension.

In our *EPHB6* KO mouse studies, *EPHB6* is deleted in all tissues^8,24^. In these mice, male KO presented normal BP, but after castration, they become hypertensive^24^. We have revealed that the vascular smooth muscle cells (VSMCs) and adrenal gland chromaffin cells (AGCCs) are *EPHB6* target cells for this BP phenotype^24^. The default function of *EPHB6* in VSMCs is to decrease their contractility. Therefore, when *EPHB6* is deleted, the opposite will occur. However, in spite of the increased VSMC contractility, the BP in the male KO mice remains normal because *EPHB6* KO in AGCCs also causes reduced ambient catecholamine secretion^24^, which neutralizes the effect of VSMC contractility increase. However, castration elevates the AGCC catecholamine secretion to a normal level^24^. This, in conjunction with high VSMC contractility in the KO mice, leads to overt hypertension in the castrated KO mice^24^. This raises an interesting hypothesis relevant to human hypertension: for a subpopulation of males with loss-of-function mutations in the *EPHB6* gene or genes in the *EPHB6* signaling pathways, they might enjoy protection against hypertension; however, if they suffer from hypogonadism at the same time, they might become hypertensive due to detrimental effects of other genetic or environmental factors.

We conducted a human genetic study to assess this hypothesis. Seven hundred and fifty hypertensive patients with hypogonadism were used as cases, and 750 normotensive hypogonadic patients as controls. SNPs in the *EPHB6*, *EFNB1* and *EFNB3* genes plus 50-kb 5′ upstream sequences of these genes were analyzed by Illumina GoldenGate custom-made arrays, protein products *EFNB1* and *EFNB3* being the ligands of EPHB6. The results show that the one SNP in the 5′ end upstream of the *EFNB3* gene start site was significantly associated with hypertension risks.

## Patients, Materials and Methods

### Patient population

A total of 4,480 male patients ≥40 years old from the Cardiology Ward, Endocrinology Ward, and Physical Examination Center of First Affiliated Hospital, College of Medicine, Zhejiang University in Hangzhou, China, were recruited for this study. They were tested for total plasma testosterone levels. Those with hypogonadism (plasma total testosterone levels <346 ng/dL, the cut-off level recommended by the International Society for the Study of the Ageing Male^31^ were retained. Among those hypogonadic individuals, 982 were diagnosed with primary hypertension and were considered as cases. The primary hypertension phenotype was defined as having a measurement of systolic pressure >140 mm Hg or diastolic pressure >90 mmHg, or having been actively treated for hypertension, excluding known conditions or medication that could cause BP increase. All other medical conditions were allowed as long as they were not the cause of hypertension. All types of medications in the last three months were allowed except those that are known to affect testosterone levels, such as testosterone replacement therapy. Seven hundred and eighty eight normotensive subjects were considered as controls. Seven hundred and fifty cases and 750 controls were selected with an attempt to match their ages as closely as possible. The means ± SE (standard error) of systolic and diastolic BP of the case and control groups are listed in Table 1. Differences of the case and control groups in potential covariate phenotype parameters (such as age, plasma testosterone level, heart rates, body mass index (BMI), serum uric acid levels and smoking status) that could be implicated in causing hypertension are listed in Table 2.

**Table 1:** Systolic and diastolic BP of cases and controls

Blood Pressure (BP)	Hypertensive Samples (Cases)			Normotensive Samples (Controls)			*P*-value for Difference of Means (*t*-test)
	N	Mean	Standard Error of Mean	N	Mean	Standard Error of Mean	
Systolic BP (mm Hg)	750	125.42	0.54	750	116.6	0.43	2.2 × 10^−16^
Diastolic BP (mm Hg)	750	76.45	0.36	750	72.06	0.32	2.2 × 10^−16^

N: number of individuals in the group.

**Table 2:** Differences in potential covariate phenotype parameters that could be implicated in causing hypertension.

Parameter	Hypertensive Samples (Cases)			Normotensive Samples (Controls)			*P*-values for Difference between Case and Control Status
	N	Mean	Standard Error of Mean	N	Mean	Standard Error of Mean	
Age (years)	750	58.80	0.26	750	57.66	0.36	1.04 × 10^−2^
Plasma Testosterone Levels (ng/dL)	750	259.76	2.16	750	264.75	2.24	ns
Heart Rate (beats per minute)	748	69.84	0.33	748	71.36	0.34	1.80 × 10^−3^
Body Mass Index (kg/m^2^)	722	25.94	0.12	705	24.71	0.12	1.91 × 10^−12^
Serum Uric Acid (μmol/L)	736	371.69	3.55	737	352.76	3.55	2.0 × 10^−4^
Number of Smokers/Number of the individuals of the group (%)		487/750 (64.9)			468/750 (62.4)		ns

ns: not statistically significant at *p* = 0.05.

*P*-values were determined by simple logistic regression of individual parameters against case versus control status.

### Ethics statement

This human genetic study was approved by the Ethics Committee of the First Affiliated Hospital of Zhejiang University (No. 2013-145), and was carried out in accordance with guidelines of the Committee. Informed consent was obtained from all the subjects recruited in this study. All experiments were conducted in accordance with other relevance guidelines and regulations of the local government.

### Blood sample collection and plasma total testosterone measurements

Venous blood samples were drawn from all subjects after an overnight fast of at least eight hours. Five mL of blood was collected into vacuum tubes with the anticoagulant EDTA-K^+^ and centrifuged at the site of collection within one hour. Cell pellets were frozen until DNA extraction.

Plasma total testosterone levels were measured with Siemens Immulite 2000 Total Testosterone Kits on Siemens Immulite 2000 Immunoassay Analyzer according to the manufacturer’s protocols.

### DNA extraction and purification

Sample DNA was extracted using DNeasy Blood & Tissue Kit (Cat. 69506, QIAGEN, Hilden, Germany) according to the manufacturer’s instructions. Purified DNA quantity and quality were assessed by Qubit^®^2.0 Fluorometer (Q32866, Invitrogen, Carlsbad CA, USA) and 1% agarose gel electrophoresis. Samples with DNA quantity ≥2 µg and optical density (OD) 260/280 = 1.8–2.0 were submitted to SNP assay.

### Candidate tag SNPs

Chromosomal regions containing sequences from the candidate genes *EPHB6*, *EFNB1* and *EFNB3* were identified from the UCSC Genome Browser^32^ using Build 37/hg19 Human Genome Assembly, and their SNPs were used for genotyping or imputation. An additional 50 kb of DNA sequence 5′ upstream of each gene were also included in order to examine potential gene regulatory elements. Tag SNPs within these regions were chosen using the Tagger program^33^ with the Han Chinese in Beijing (CHB) linkage-disequilibrium (LD) data. For *EPHB6*, tag SNPs in the first 2.4 kb of the *EPHB6* gene between positions 142552792 and 142555251, which ended in the first exon, plus 50-kb 5′ sequence was selected for genotyping. Tag SNPs were chosen with to represent proxy SNPs with a minimum linkage disequilibrium (LD) value coefficient of determination (*r*^*2*^) >0.80 and minor allele frequency >0.05. Candidate tag SNPs were then submitted for analysis by Illumina Software for compatibility with the GoldenGate multiplexing process. Alternative Tag SNPs were chosen for those Tag SNPs determined to be incompatible with the multiplexing technology and the new Tag SNPs were then re-submitted for a new Illumina software analysis to determine the compatibility of the new set of SNPs including previously compatible SNPs and the new alternative Tag SNPs. This process continued in an iterative manner until all Tag SNPs were found to be compatible for use by the multiplexing technology. Ultimately, seven tag SNPs were chosen for *EPHB6*, seven tag SNPs for *EFNB1*, and nine tag SNPs for *EFNB3* (Table 3). Their chromosomal positions and minor allele frequencies are also presented (Table 3). *Bonferroni*-corrected critical significance level (*p*_*crit*_) were calculated for SNP and hypertension associations assuming tag SNPs represent independent statistical tests when performing association analysis across the regions: *p*_*crit*_ = 0.0071 for *EPHB6* alone (*p*_crit_ = 0.05/7); *p*_*crit*_ = 0.0071 for *EFNB1* alone (*p*_crit_ = 0.05/7); *p*_*crit*_ = 0.0056 for EFNB3 alone (*p*_*crit*_ = 0.05/9), and *p*_*crit*_ = 0.0024 for the experiment-wide analysis of all the three genes combined (*p*_*crit*_ = 0.05/21).

**Table 3:** Selected Genotyped Tag SNPs for EFNB1, EFNB3 and EPHB6 gene regions.

GENE	SNP	Chromosome	Position Build 37/hg19	Minor Allele Frequency from CHB HapMap
EPHB6	rs3134904	7	142503276	0.190
EPHB6	rs3134905	7	142503362	0.214
EPHB6	rs11773714	7	142523468	0.144
EPHB6	rs9986701	7	142534935	0.054
EPHB6	rs10261171	7	142536157	0.211
EPHB6	rs12537777	7	142536951	0.071
EPHB6	rs1009848	7	142555251	0.083
EFNB1	rs241386	23	68043534	0.310
EFNB1	rs697500	23	68045068	0.365
EFNB1	rs638408	23	68051015	0.349
EFNB1	rs877817	23	68053122	0.119
EFNB1	rs877818	23	68053507	0.103
EFNB1	rs16990746	23	68057610	0.111
EFNB3	rs1641511	17	7559677	0.446
EFNB3	rs1050540	17	7560742	0.071
EFNB3	rs1050541	17	7560835	0.399
EFNB3	rs12951053	17	7577407	0.333
EFNB3	rs9895829	17	7578679	0.101
EFNB3	rs7640	17	7606722	0.489
EFNB3	rs12941981	17	7608462	0.259
EFNB3	rs3744263	17	7613708	0.405
EFNB3	rs7141	17	7614601	0.488

CHB: Chinese Han, Beijing.

### Tag SNP genotyping

The tag SNPs were genotyped by the Shanghai Biotechnology Corporation using the Illumina GoldenGate genotyping platform according to the manufactures instructions. Those SNPs with a call rate less than 90% were filtered out and not analyzed.

### Association analysis

The genotyped SNPs were tested for Hardy-Weinberg equilibrium and were then used to impute additional SNPs within the region covered by the tag SNPs using the IMPUTE2 program^34^ and the 1000 Genomes Phase 3 East Asian LD dataset^35^. Thirty-eight additional SNPs were imputed for the *EFNB1* region, 124 for the *EFNB3* region and 204 for the *EPHB6* region. The imputed and the genotyped tag SNPs were then analyzed for association with hypertensive versus normotensive status across all three regions employing the PLINK^36^ program, and using a logistic regression model with or without covariates. Each SNP was assigned a reference allele and an alternative allele. The number of reference alleles in each individual was entered as its genotype value. These were summed over all cases and controls and entered into the logistic regression equation as the genotype term. This is analogous to an additive genetic model. Therefore, odds ratios refer to the reference allele which is noted in the tables.

For the X-linked *EFNB1* gene, the SNP imputation also utilized IMPUTE2, which handles imputation for both sex and autosomal chromosomes. The program essentially has two steps. In the first step the haplotypes are estimated from the genotype values and allele frequencies using a Bayesian expectation-maximization iterative procedure. The second step determines the alleles of non-genotyped SNPs based upon their occurrence on the estimated haplotypes as determined by the 1000 genome project data for Chinese Asian populations. The haplotype determination step will have a small error associated with it for autosomal and for X-linked genes in females. However, males are hemizygous for X-linked genes, so the genotype sequences in males are the haplotypes. IMPUTE2 treats X-linked genes in males as homozygotes for the purpose of haplotype estimation, so for males (the patients in our cohort are all males), there is no error in the haplotype estimation step. Hence, X-linked haplotype determination is error-free in our case, and is thus more accurate. However, for the X-linked genes in males, there is only half the number of alleles to count for the association analysis compared to autosomal genes. As a consequence, we have a smaller sample size for X-linked SNPs compared to autosomal SNPs, and there will be higher *p*-values for an association of the same effect size.

## Results

In this study, we first screened 4,480 male patients, and retained only those with hypogonadism, diagnosed according to the criteria of the International Society for the Study of Ageing Males (plasma testosterone levels <346 ng/dL). It is to be noted that although the mean BP (systolic as well as diastolic) was higher in the cases than the controls as expected (Table 1), they did not reach the hypertension diagnostic criteria, *i*.*e*., systolic pressure >140 mmHg and/or diastolic pressure >90 mmHg. This is because the majority of the cases (93.7%) were previously diagnosed and were under anti-hypertension medication; their BP was largely under control to be within the normal range.

Means or percentages of various additional parameters, age, plasma testosterone levels, heart rates, body mass index (BMI), serum uric acid levels and smoking status that might affect hypertension status are presented in Table 2. Simple logistic regression of each of these individual parameters against case versus control status was conducted to assess the significance of the differences in these parameters between cases and controls. The *p*-values of these tests are shown in Table 2. Despite our efforts to match the ages of the cases and controls, there was still a slight but statistically significant difference in mean age (*p* = 0.0104). The plasma testosterone levels of the cases were also slightly lower than those of the controls, but the difference was not significant. The heart rates of the cases were significantly slower (*p* = 0.0018) than those of the controls. This is likely due to reflexive feedback regulation of the higher BP in the cases. The cases had significantly higher BMI than the controls (*p* = 1.91 × 10^−12^). The higher BMI could be a contributing factor to hypertension in the cases. Finally, the cases had significantly higher serum uric acid levels than the controls (*p* = 0.0002). This probably reflects kidney damage caused by hypertension, because we only recruited patients with primary hypertension, and none of the cases was hypertensive due to primary renal diseases.

**Figure 1:**
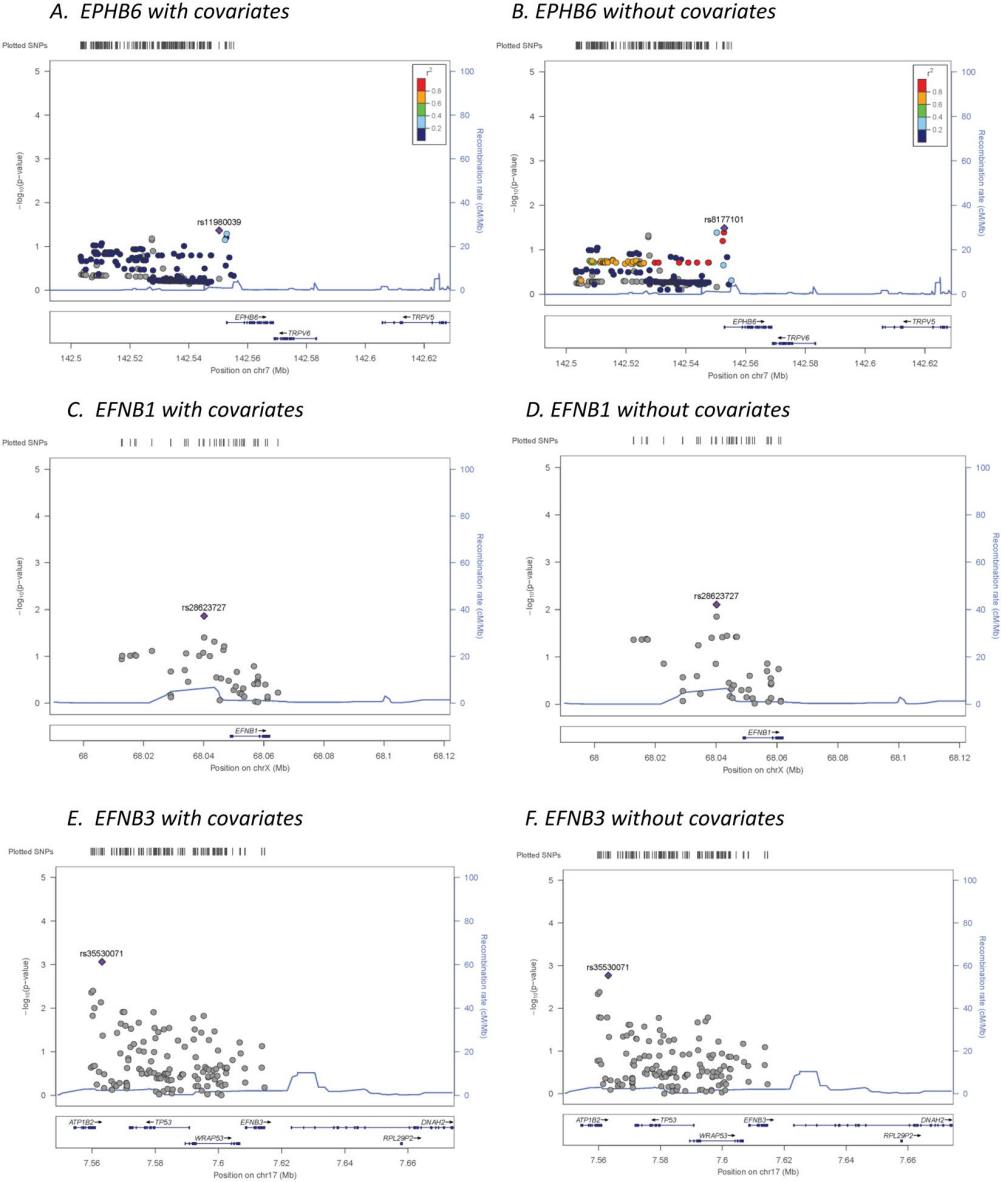
LocusZoom plots for association of all SNPs assayed across EPHB6, EFNB1 and EFNB3 gene regions for regression models with and without covariates. Left Y-axis: -Log_10_ (*p*-value) of the SNP association with hypertension. Right Y-axis: Recombination rate (cM/Mb). X-axis: position of SNPs and EFNB3 gene in chromosome 7. Color bar: *r*^*2*^ value.

The potential covariate parameters listed in Table 2 were used in a stepwise multiple logistic regression against case and control status. The results (Table 4) show that all of these parameters, when combined in a multiple regression model, were significantly different between cases and controls, except testosterone levels, which were nearly significant with a *p*-value slightly above 0.05 (*p* = 0.0506), and smoking status, which was not significant. We thus used the significant and near-significant parameters, *i*.*e*., age, testosterone level, heart rate, BMI and serum uric acid levels, as covariates in our following genetic association analysis in order to eliminate the possibility of confounding them with genotype association to hypertension, even though we consider that some of them might be consequences rather than causes of hypertension. The genetic association was tested using logistic regression models with or without the covariates. The results of the association study of all genotyped tag SNPs and imputed SNPs across all three tested gene regions (*EPHB6*, *EFNB1*, and *EFNB3*) are presented in the supplementary materials (Supplementary Table 1). Reference alleles used in coding the genotypes, alternative alleles, minor alleles, minor allele frequencies, information scores for the imputed SNPs, odds ratios of association with respect to the reference allele, standard error of odds ratios and *p*-values of association models with or without covariates are presented for each SNP. Locus zoom plots for association across the three gene regions are presented in Figure 1 for both models with and without covariates.

**Table 4:** Significance of parameters used as covariates in a stepwise multiple regression model.

Parameter	Coefficient	Std. Error	Z-score	*P*-value
(Intercept)	−3.65882	0.882338	−4.15	3.37 × 10^−05^
Age	0.02668	0.006828	3.91	9.32 × 10^−05^
Testosterone Levels	−0.00183	0.000937	−1.95	5.06 × 10^−02^
Heart Rate	−0.01937	0.006275	−3.09	2.03 × 10^−03^
Body Mass Index	0.13036	0.018895	6.90	5.23 × 10^−12^
Serum Uric Acid	0.00185	0.000595	3.11	1.85 10^−03^

In general, the *p*-values of association for the covariate models displayed similar general patterns of association but were slightly lower than in the models that did not incorporate the covariates for many SNPs. This suggests that the effects of covariates and genotypes were somewhat but not completely independent. No SNPs approached significance in the tested *EPHB6* or *EFNB1* gene regions in models with or without covariates; however, one imputed SNP located 5′ of ENFB3, *rs35530071* (Chromosome 17 position 7563124 Build 37/hg 19, *p* = 0.00087 for the covariate model and *p* = 0.00170 for the model without covariates; Table 5) reached 3-gene experiment-wide significance (*p*_crit_ ≤ 0.0024). Two other nearby imputed SNPs, *rs73*2*46870* and *rs61209339* (Chromosome 17 positions 7559777 and 7560279) reached significance for the *Bonferroni* correction for the single gene-only analysis (9 tag SNPs *p*_*crit*_ = 0.0055). The most significant nearby genotyped SNP was *rs1050540* (Chromosome 17 positon 7560742, *p* = 0.0099 for the covariate model and *p* = 0.01663 for the model without covariates), which did not reach *Bonferroni*-corrected significance level.

**Table 5:** Selected results of logistic regression association tests for EFNB3 with and without covariates.

SNP	SNP Genotyped or Imputed	Position on Chrom 17 Build 37/hg19	Reference Allele	Alternative Allele	Minor Allele	Minor Allele Frequency	Imputation Information Score	Model with Covariates			Model without covariates		
								Odds Ratio	SE of Odds Ratio	P-value of Association	Odds Ratio	SE of Odds Ratio	P-value of Association
rs1641511	Genotyped	7559677	G	A	G	0.39	NA	0.914	0.076	0.23180	0.903	0.074	0.17040
**rs73246870**	Imputed	7559777	C	G	G	0.05	0.772	1.769	0.200	**0**.**00441**	1.752	0.198	**0**.**00463**
rs370458118	Imputed	7560153	C	CTT	CTT	0.48	0.658	0.799	0.092	0.01498	0.805	0.091	0.01626
**rs61209339**	Imputed	7560279	G	T	T	0.05	0.782	1.782	0.201	**0**.**00400**	1.766	0.199	**0**.**00417**
rs1050533	Imputed	7560294	C	T	T	0.50	0.580	0.886	0.098	0.21570	0.876	0.096	0.16670
rs1050540	Genotyped	7560742	C	T	T	0.09	NA	1.411	0.134	0.00993	1.370	0.132	0.01663
rs1050541	Genotyped	7560835	T	G	G	0.36	NA	1.100	0.076	0.21200	1.099	0.075	0.20460
rs1641510	Imputed	7561496	G	A	G	0.37	0.732	0.950	0.090	0.56660	0.954	0.088	0.59610
rs35899238	Imputed	7562174	T	G	G	0.06	0.678	1.228	0.194	0.28990	1.156	0.190	0.44370
rs28461213	Imputed	7562790	A	G	G	0.11	0.841	1.421	0.131	0.00737	1.362	0.129	0.01646
**rs35530071**	Imputed	7563124	C	CT	CT	0.09	0.612	1.747	0.168	**0**.**00087**	1.675	0.164	**0**.**00170**
rs2908807	Imputed	7563354	T	C	T	0.46	0.754	0.840	0.086	0.04288	0.845	0.085	0.04699
rs72485505	Imputed	7563826	AC	A	A	0.06	0.609	1.214	0.199	0.32890	1.144	0.194	0.48720
rs2908806	Imputed	7563827	C	A	C	0.12	0.437	1.073	0.173	0.68330	1.041	0.171	0.81530
rs12600850	Imputed	7566133	A	G	G	0.35	0.853	1.062	0.085	0.47540	1.056	0.083	0.51260
rs62062581	Imputed	7566274	T	G	G	0.27	0.928	1.029	0.087	0.74700	1.039	0.086	0.65950
rs78337160	Imputed	7566326	CT	C	C	0.26	0.931	1.028	0.087	0.74940	1.038	0.086	0.66280
rs9674772	Imputed	7566979	A	G	G	0.34	0.857	1.051	0.085	0.56190	1.045	0.084	0.60160
rs12940247	Imputed	7567703	A	G	A	0.40	0.727	0.956	0.089	0.61050	0.958	0.087	0.62400
rs34289020	Imputed	7567902	C	CT	CT	0.39	0.836	1.191	0.084	0.03700	1.178	0.082	0.04609
rs201214141	Imputed	7568712	A	G	G	0.24	0.902	1.060	0.092	0.52570	1.073	0.090	0.43360
rs55745760	Imputed	7568925	C	T	C	0.48	0.679	0.865	0.091	0.10930	0.870	0.089	0.11500
rs71159520	Imputed	7569109	C	CA	CA	0.12	0.714	1.362	0.136	0.02271	1.308	0.133	0.04371
rs8073498	Imputed	7569698	A	C	C	0.11	0.784	1.402	0.135	0.01230	1.347	0.132	0.02438
rs9893249	Imputed	7570189	T	C	C	0.11	0.783	1.401	0.135	0.01232	1.347	0.132	0.02428
rs34569991	Imputed	7570578	TA	T	TA	0.15	0.548	0.724	0.144	0.02532	0.745	0.142	0.03762

Results of logistic regression association tests for SNPs located 48,843 bp to 37,942 bp 5′ upstream of the EFNB3 gene are shown. SNPs having *p*-values of association with hypertension below the single-gene critical *Bonferroni* significance level of 0.0055 for 11 LD blocks within the EFNB3 gene are in bold. SNP *rs35530071* with *p*-value of association below the experiment-wise critical *Bonferroni* significance level of 0.0024 is in bold.

NA: not applicable.

These 4 SNPs are all located in the far 5′ region upstream of the start site for *EFNB3*. SNP associations with hypertension within this region are shown in Table 5.

To assess the impact of each of the covariates on the significant SNP associations with hypertension, we re-analyzed the multiple logistic regression associations for the above-mentioned four SNPs using the R software^37^, which provides the estimates and *p*-values of significance for all covariate terms as well as for the genotype terms. These values are presented in Table 6. The blood testosterone term had a negative coefficient indicating that it is negatively correlated with hypertension, as expected. It became slightly more significant when the multiple regression model included the genotype terms, the *p*-values going from just above 0.05 without a genotype term (Table 4) to near 0.041–0.045 (Table 6). The general orders of magnitude of significance remained the same for the other covariates for models with and without the genotype term, with BMI being the most significant covariate.

**Table 6:** Estimates of logistic coefficients, standard errors of estimates, Z scores and *p*-values of association for all terms of the genotype with covariates logistic multiple regression models for the four SNPs 5′ of the EFNB3 gene region with the lowest genotype *p*-values of association as produced by the R software.

SNP	Term	Coefficient	Std. Error	Z score	*P*-value
rs3550071	Intercept	−3.4207	0.8833	−3.873	0.000108
	Genotype	−0.5507	0.1749	−3.149	0.001639
	Age	0.0253	0.0068	3.696	0.000219
	Testosterone	−0.0019	0.0009	−2.045	**0**.**040903**
	Uric Acid	0.0020	0.0006	3.267	0.001087
	BMI	0.1285	0.0189	6.795	1.09 × 10^−11^
	Heart Rate	−0.0197	0.0063	−3.125	0.001775
rs61209339	Intercept	−0.3070	0.2014	−1.525	0.127592
	Genotype	−0.1314	0.0475	−2.767	0.005741
	Age	0.0060	0.0016	3.782	0.000162
	Testosterone	−0.0004	0.0002	−2.003	**0**.**045372**
	Uric Acid	0.0005	0.0001	3.272	0.001095
	BMI	0.0293	0.0041	7.124	1.67 × 10^−12^
	Heart Rate	−0.0044	0.0014	−3.067	0.002205
rs73246870	Intercept	−0.3065	0.2014	−1.522	0.128348
	Genotype	−0.1298	0.0474	−2.737	0.00627
	Age	0.0060	0.0016	3.782	0.000162
	Testosterone	−0.0004	0.0002	−2.004	**0**.**045219**
	Uric Acid	0.0005	0.0001	3.272	0.001093
	BMI	0.0293	0.0041	7.125	1.67 × 10^−12^
	Heart Rate	−0.0044	0.0014	−3.072	0.002168
rs1050540	Intercept	−0.2959	0.2015	−1.468	0.142241
	Genotype	−0.0851	0.0324	−2.622	0.00884
	Age	0.0060	0.0016	3.789	0.000158
	Testosterone	−0.0004	0.0002	−2.037	**0**.**041878**
	Uric Acid	0.0005	0.0001	3.276	0.001079
	BMI	−0.0045	0.0014	−3.15	0.001669
	Heart Rate	0.0293	0.0041	7.124	1.68 × 10^−12^

## Discussion

Our previous animal study demonstrates that castrated *EPHB6* KO mice exhibit hypertension, suggesting that *EPHB6* and/or its signalling pathways are involved in testosterone-dependent blood pressure control. To assess the relevance of this phenotype to human hypertension, in this study we investigated the association of SNPs in the *EPHB6* gene, and in the genes of its two ligands, *EFNB1* and *EFNB3*, with hypertension in Han Chinese hypogonadic males.

EPHB6 has three potential ligands, *i*.*e*., EFNB1, EFNB2 and EFNB3. The deletion of EPHB6, EFNB1 and EFNB3 all result in hypertension in mice, while the deletion of EFNB2 leads to an opposite phenotype, *i*.*e*., hypotension. Thus, we speculated that EFNB1 and EFNB3 are more relevant to EPHB6 signalling for the hypertension phenotype, and hence they were included in our current hypertension association study. Regulatory elements (enhancers or repressors) are often present in the 5′ region of genes. So SNPs in the 50-kb regions upstream of these genes were added for the analysis. While no SNPs in analyzed *EPHB6* or *EFNB1* regions were significantly associated with hypertension, three markers (one at a 3-gene experiment-wide significance level, and two at an EFNB3 single-gene significance level) located 5′ upstream of the *EFNB3* gene start site were significantly associated with hypertension in this population even after correction for all of the covariates, which were also all significantly or near-significantly associated with hypertension. A quantitative analysis of the association of the SNPs of these three gene regions with the BP levels, after adjusting for medication (by adding 15 mmHg to SP and 10 mmHg to DP), showed similar results (data now shown) as the qualitative analysis.

Why were SNPs in the *EFNB3* gene but not the *EPHB6* gene significantly associated with hypertension in hypogonadic males, in spite of the *EPHB6* KO leading to hypertension in castrated mice? In mice, when we delete EPHB6, its signalling, including the forward signalling from EFNBs to EPHB6, and reverse signalling from EPHB6 to EFNBs, could be compromised. Any defect in these signalling pathways could lead to the hypertension phenotype, while EPHB6 itself might not be critically important. Indeed, EFNB3 deletion also causes hypertension^26^, suggesting that at least some BP-related phenotype in *EPHB6* KO mice is involving EFNB3 via signalling.

Another possible explanations are that (1) significantly associated SNPs might lie in the remaining 13.6 kb of the *EPHB6* gene or even in the 3′ flanking sequence of *EPHB6* gene, which are yet to be tested; or (2) while the lack of EPHB6 function leads to hypertension as demonstrated by the gene knockout results in the mouse model, it may simply be that there are no functional polymorphisms present within the analysed *EPHB6* gene region in this human population that could result in loss of function of the gene.

We did a search of eQTL of the three most significant SNPs in the *EFNB3* gene region, *i*,*e*., *rs35530071* (located at 45,396 bp 5′ upstream of the *EFNB3* gene start site), *SNP rs73246870* (located 48,743 bp 5′ upstream of *EFNB3* gene start site) and *rs61209339* (located 48,241 bp 5′ upstream of *EFNB3* gene start site, using GRex^38^. The database did not show any significant association of these SNPs with EFNB3 expression changes in any tissue, suggesting that these SNPs are unlikely functional mutations, or such expression data are not yet available. These SNPs appear to be in high LD with each other, and thus indicate the presence of a single functional polymorphism within this LD block located from 48.5 to 37.9 kb 5′ upstream of the *EFNB3* gene. It is not uncommon to have regulatory elements located at such a distance 5′ to the start site of the gene. For example, β-globin has an enhancer at 50-kb 5′ upstream of its start site^39^; apoB has an effective enhancer at 55-kb 5′ upstream of its start site^40^. Several genes (*e*.*g*., *FOXG1* and *MYC*) even have enhancers at a distance 1 mb or farther from the start sites^41^. In some cases, there are additional genes located between an enhancer at a large distance away and the gene it regulates. Yet the enhancer would ignore the closest promoters and favour the one situated at a distance^42^. In the nuclei, DNA is coiled, and thus an enhancer at a seemingly long linear distance from a promoter in a three-dimensional space might actually be at the proximity of the latter, due to directed or assisted looping of DNA treads^41^. In the case of *EFNB3*, *in silico* search shows that it has a highly ranked enhancer at 27.9 kb 5′ of its start site^43^. Therefore, it is entirely possible that there is another regulatory element in the LD block which spans from 48.5 to 37.9 kb 5′ of the *EFNB3* start site, despite that there are two other genes (*TP53* and *WRAP53*) between the putative regulatory element and *EFNB3*.

To prove experimentally that *EFNB3* mutations in the hypogonadic patients could indeed cause hypertension, we investigated the BP in *EFNB3* KO mice. The male *EFNB3* KO mice are normotensive^26^. The BP of the castrated WT males was decreased compared to uncastrated WT ones^26^. This is consistent with other reports that reduced testosterone level is often associated with lower BP in animal models^44^. However, in KO males, castration had no such BP-lowering effect, and as a result, the BP of the castrated KO mice was significantly higher than that of the castrated WT mice^26^. This *in vivo* result in mice suggests that male patients with *EFNB3* loss-of-function mutations could not enjoy the BP-lowering benefit of reduced testosterone levels, which could be due to either ageing or other medical conditions. For these patients, if they suffer from other detrimental genetic or environmental influences that cause BP increase, the lost protection will render them hypertensive, as hypertension is a polygenic and multifactorial disease. As a consequence, compared to individuals without such *EFNB3* loss-of-function mutations, these patients will have relatively increased hypertension risks. This is consistent with the results of our current human genetic study, in which we reveal that three *EFNB3* SNPs are significantly associated with hypertension in hypogonadic patients.

Similar studies in patient cohorts from different ethnic backgrounds are warranted to generalize the conclusion that loss-of-function mutations in the EPHB6 signalling pathway molecules is a relative hypertension risk, and that such risk will only be materialized after testosterone levels are reduced.

## Electronic supplementary material

Supplementary data
