# Supplementary material for: Analysis of the association of EPHB6, EFNB1 and EFNB3 variants with hypertension risks in males with hypogonadism
Source: Sci Rep. 2018 Sep 27;8:14497. doi: 10.1038/s41598-018-32836-x (PMC6160468; doi:10.1038/s41598-018-32836-x)
Supplement: Supplementary file 1 — Supplementary data [file 41598_2018_32836_MOESM1_ESM.docx]

**Supplementary Materials**

**Analysis of the association of EPHB6, EFNB1 and EFNB3 variants with hypertension risks in males with hypogonadism**

^1#^Tao Wu, ^1#^Bi-Qi Zhang, ^2^John Raelson, ^3^Yu-Mei Yao, ^1^Huan-Dong Wu, ^1^Zao-Xian Xu, ^2^Francois-christophe Marois-blanchet, ^2^Muhammad Ramzan Tahir, ^2,4^Yujia Wang, ^2^W. Edward Bradley, ^2^Hongyu Luo, ^2,5*^Jiangping Wu, ^6*^Jian-Zhong Sheng, and ^1*^Shen-Jiang Hu

Running title: EPHB6, EFNB1 and EFNB3 mutations in hypertension

From ^1^Institute of Cardiology, First Affiliated Hospital, College of Medicine, Zhejiang University, Hangzhou, 310003, China; ^2^Research Centre and ^5^Nephrology Service, Centre hospitalier de l’Université de Montréal (CHUM), Montreal, Quebec H2X 0A9, Canada; ^3^Department of Cardiology, Third Affiliated Hospital of Zhejiang Chinese Medical University, Hangzhou, 310005, China; ^4^Children’s Hospital, Zhejiang University School of Medicine, Hangzhou, Zhejiang, China 310003; ^6^Department of Pathology and Physiopathology, College of Medicine, Zhejiang University, Hangzhou, 310005, China

*Address correspondence to: Dr. Jiangping Wu, CHUM Research Center (CRCHUM), 900 Saint-Denis Street, Rm. R12.428, Montreal, Quebec H2X 0A9, Canada, telephone: (514) 890-8000 Extension 25164, Fax: (514) 412-7944, e-mail: [jianping.wu@umontreal.ca](mailto:jianping.wu@umontreal.ca); or Jian-Zhong Sheng, Department of Pathology and Physiopathology, College of Medicine, Zhejiang University, Hangzhou, 310005, China telephone: 86-571-88208803, e-mail: shengjz@zju.edu.cn; or Shen-Jiang Hu, Institute of Cardiology, First Affiliated Hospital, Zhejiang University Medical College, Hangzhou, 310003, China

^#^ These two authors contributed equally to this work.

*Supplementary Table 1. Complete results of logistic association regression tests with and without covariates of individual SNPs across the tested EPHB6, EFNB1 and EFNB3 gene regions*

A. *EFNB1 region (gene plus 50-kb 5’sequence)*

NA = not applicable

*B. EFNB3 region (gene plus 50-kb 5’ sequence)*

*B. EFNB3 region (gene plus 50-kb 5’ sequence) (cont’d)*

Results of logistic regression association tests for SNPs in the tested region of the EFNB3 gene are shown. SNPs having *p*-values of association with hypertension below the single gene critical *Bonferroni* significance level of 0.0055 for 11 LD blocks within the EFNB3 gene are in bold. SNP *rs35530071* with *p*-value of association below the experiment-wise critical *Bonferroni* significance level of 0.0024 is highlighted in grey.

NA: not applicable.

*C. EPHB6 region (first 2.5 kb of the gene plus 50-kb 5’sequence)*

NA: not applicable.

*C. EPHB6 region (first 2.5 kb of the gene plus 50-kb 5’ sequence) (cont’d)*

*C. EPHB6 region (first 2.5 kb of the gene plus 50-kb 5’ sequence) (cont’d)*
